# Survivin family proteins as novel molecular determinants of doxorubicin resistance in organotypic human breast tumors

**DOI:** 10.1186/bcr3666

**Published:** 2014-05-30

**Authors:** Alice Faversani, Valentina Vaira, Giacomina P Moro, Delfina Tosi, Alessia Lopergolo, David C Schultz, Dayana Rivadeneira, Dario C Altieri, Silvano Bosari

**Affiliations:** 1 Division of Pathology, Fondazione IRCCS Ca’ Granda Ospedale Maggiore Policlinico, via F. Sforza 35, Milan 20122, Italy; 2 Breast Unit, Ospedale San Paolo, Milan 20142, Italy; 3 Department of Health Science, Ospedale San Paolo, University of Milan, via Antonio di Rudinì 8, Milan 20142, Italy; 4 Molecular Pharmacology Unit, Department of Experimental Oncology and Molecular Medicine, Fondazione IRCCS-Istituto Nazionale Tumori, via Giacomo Venezian 1, Milan 20133, Italy; 5 Center for Chemical Biology and Translational Medicine, The Wistar Institute, 3601 Spruce Street, Philadelphia, PA 19104, USA; 6 Molecular and Cellular Oncogenesis Program, The Wistar Institute, 3601 Spruce Street, Philadelphia, PA 19104, USA; 7 Department of Pathophysiology and Transplantation, University of Milan, Via Festa del Perdono 7, Milan 20122, Italy

## Abstract

**Introduction:**

The molecular determinants of breast cancer resistance to first-line anthracycline-containing chemotherapy are unknown.

**Methods:**

We examined the response to doxorubicin of organotypic cultures of primary human breast tumors *ex vivo* with respect to cell proliferation, DNA damage and modulation of apoptosis. Samples were analyzed for genome-wide modulation of cell death pathways, differential activation of p53, and the role of survivin family molecules in drug resistance. Rational drug combination regimens were explored by high-throughput screening, and validated in model breast cancer cell types.

**Results:**

Doxorubicin treatment segregated organotypic human breast tumors into distinct Responder or Non Responder groups, characterized by differential proliferative index, stabilization of p53, and induction of apoptosis. Conversely, tumor histotype, hormone receptor or human epidermal growth factor receptor-2 (HER2) status did not influence chemotherapy sensitivity. Global analysis of cell death pathways identified survivin and its alternatively spliced form, survivin-ΔEx3 as uniquely overexpressed in Non Responder breast tumors. Forced expression of survivin-ΔEx3 preserved cell viability and prevented doxorubicin-induced apoptosis in breast cancer cell types. High-throughput pharmacologic targeting of survivin family proteins with a small-molecule survivin suppressant currently in the clinic (YM155) selectively potentiated the effect of doxorubicin, but not other chemotherapeutics in breast cancer cell types, and induced tumor cell apoptosis.

**Conclusions:**

Survivin family proteins are novel effectors of doxorubicin resistance in chemotherapy-naive breast cancer. The incorporation of survivin antagonist(s) in anthracycline-containing regimens may have improved clinical activity in these patients.

## Introduction

Despite considerable progress in the molecular characterization [1], and treatment [2] of breast cancer, drug-resistant disease remains a common occurrence, often heralding high morbidity and mortality due to metastatic progression. The molecular underpinnings of treatment-resistant breast cancer, which includes insensitivity to antiestrogen regimens [3], and refractoriness to epidermal growth factor receptor-2 (HER2) inhibitors [4], have been intensely investigated, and linked to aberrant receptor tyrosine kinase signaling [5], enhanced drug efflux mechanisms [6], and defective immune recognition [7]. Although several strategies have been examined to restore treatment sensitivity in these settings [8,9], resistance to the most common, first-line anthracycline-containing chemotherapy [10] continues to represent a significant challenge [11], with limited, if any, ‘actionable’ molecular targets to restore drug sensitivity.

In this context, resistance to apoptosis, or programmed cell death, is a common occurrence of treatment-resistant malignancies [12], involving deregulated expression of cell death modulators of the Bcl-2 [13], or inhibitor of apoptosis (IAP) [14] gene family, including survivin [15]. In chemotherapy-resistant breast cancer, these pathways further compound other aberrant mechanisms of cell survival, including loss of the *PTEN* tumor suppressor gene [16], reactivation of phosphatidylinositol 3-kinase (PI3K)/mammalian target of rapamycin (mTOR) signaling [17], expansion of cancer-initiating, progenitor-like cells [18], and increased production of vascular endothelial cell growth factor (VEGF) [19]. Although many of these pathways contain ‘actionable’ molecular targets, a key challenge in dissecting their role in drug resistance is the paucity of reliable disease model(s) that recapitulate the complexity of the human disease, while preserving the integrity of the tumor microenvironment, as a recognized disease driver in breast cancer [20]. To overcome this barrier, short-term *ex vivo* cultures of organotypic primary human tumors may provide a flexible translational platform, suitable to evaluate the impact of deregulated signaling pathways [21], and molecular therapies [22], under conditions that preserve tumor architecture [20].

In this study, we used fresh organotypic tissue cultures from treatment-naïve human breast tumors to explore the molecular requirements of anthracycline resistance [10]. We identified a discrete subgroup of doxorubicin-insensitive, that is Non Responder tumors, characterized by high proliferative index, impaired p53 responses and resistance to apoptosis. In turn, molecular analyses demonstrated that aberrant overexpression of survivin family proteins [15] is required to maintain the Non Responder phenotype, opening fresh opportunities for rational combination regimens to restore anthracycline sensitivity in these patients.

## Methods

### Patient cohort

Primary human breast tumors were obtained from 33 patients who underwent surgery for therapeutic purposes at San Paolo Hospital (Milan, Italy). The clinicopathologic and molecular characteristics of the patients analyzed in this study are presented in Table 1. Patients who received neoadjuvant chemotherapy and/or radiotherapy were excluded from the study. Informed consent was obtained from all patients and the study was approved by the Institutional Review Board of the San Paolo Hospital.

**Table 1:** **Clinicopathological and molecular characteristics of breast cancers analyzed (n = 33)**^**a**^

**Case**	**Histotype**^**b**^	**T**	**N**	**G**^**c**^	**ER**	**PR**	**HER2 status**^**d**^	**Ki-67**^**e**^	**p53**	**Doxo class**^**f**^
BR1	IDC	pT1c	N1a	G2	70%	70%	Neg	10%	Wt	Responder
BR2	IDC	pT2	pNx	G2	90%	70%	Neg	15%	R280G	Responder
BR3	IDC	pT3	N1a	G2	80%	Neg	Neg	20%	Wt	Non Responder
BR4	IDC	pT1c	N0	G2	80%	10%	Pos	10%	Wt	Non Responder
BR5	IDC	pT2	N3a	G3	80%	90%	Neg	20%	Wt	Non Responder
BR6	PC	pT2	N2a	G3	90%	5%	Neg	10%	Wt	Responder
BR7	DLC	pT3	N1	G3	90%	5%	Neg	30%	Wt	Responder
BR8	IDC	pT1c	N1a	G3	80%	70%	Neg	25%	Wt	Responder
BR9	IDC	pT1c	N0	G2	90%	70%	Neg	20%	Wt	Responder
BR10	IDC	pT2	Nx	G2	90%	95%	Neg	15%	Wt	Responder
BR11	PC	pT2	N0	G1	95%	95%	Neg	10%	Wt	Responder
BR12	IDC	pT1c	N0	G3	95%	60%	Neg	40%	Wt	Non Responder
BR13	IDC	pT1c	N0	G2	90%	85%	Neg	15%	Wt	Non Responder
BR14	ILC	pT3	N3a	G3	25%	40%	Pos	20%	Wt	Non Responder
BR15	IDC	pT2	N0	G3	95%	20%	Neg	10%	Wt	Responder
BR16	IDC	pT1c	N0	G2	95%	Neg	Neg	5%	Wt	Non Responder
BR17	IDC	pT1c	N0	G2	95%	95%	Neg	20%	Wt	Non Responder
BR18	IDC	pT3	N3	G3	Neg	Neg	Neg	45%	Wt	Responder
BR19	IDC	pT2	N2	G3	90%	60%	Pos	90%	Wt	Non Responder
BR20	IDC	pT2	N3a	G3	90%	2%	Pos	10%	Wt	Responder
BR21	IDC	pT2	Nx	G3	90%	80%	Neg	30%	Wt	Non Responder
BR22	IDC	pT1c	N0	G1	90%	90%	Neg	5%	Wt	Responder
BR23	IDC	pT1c	Nx	G2	90%	40%	Neg	20%	Wt	Responder
BR24	IDC	pT1c	Nx	G2	95%	95%	Neg	5%	Wt	Responder
BR25	IDC	pT2	N2a	G1	90%	75%	Neg	5%	K132N	Responder
BR26	IDC	pT2	N1a	G2	90%	70%	Neg	10%	Wt	Non Responder
BR27	IDC	pT1c	N0	G3	90%	90%	Neg	20%	Wt	Non Responder
BR28	IDC	pT2	N1a	G3	90%	10%	Neg	30%	Wt	Responder
BR29	IDC	pT1c	N0	G3	90%	Neg	Neg	25%	Wt	Responder
BR30	IDC	pT1c	N1a	G1	95%	95%	Neg	5%	Wt	Responder
BR31	IDC	pT1c	N0	G3	Neg	Neg	Neg	40%	Wt	Non Responder
BR32	IDC	pT2	N1a	G3	95%	70%	Neg	25%	R248W	Non Responder
BR33	IDC	pT3	N3a	G2	90%	80%	Neg	10%	Wt	Responder

^a^Tumor stage according to TNM staging systems, grade, hormone or HER2 receptor status, tumor proliferation (percentage of Ki-67-positive cells), and p53 gene mutations are indicated along with our biological class related to the *ex vivo* doxorubicin treatment. All patients were M0, that is, no distant metastases were present at diagnosis. ^b^IDC, invasive ductal carcinoma; PC, papillary carcinoma; DLC, ductal and lobular carcinoma; ILC, invasive lobular carcinoma.^c^G, tumor grade. ^d^HER2 status was determined according to American Society of Clinical Oncology guidelines (2007). ^e^Ki-67 index as determined at diagnosis (approximate to the nearest 5%). ^f^Doxorubicin class was assigned to breast cancers depending on the decrease of proliferating cells upon *ex vivo* doxorubicin treatment of at least 50% (Responders) or the maintenance of Ki-67-positive cells similar to control treated cultures (Non Responders).

### Tissue slice preparation

Tissue processing was performed within 20 minutes after surgical resection. Tissue slices (400 μm thick) were obtained through serial cutting of the individual samples using a Vibratome VT1200 (Leica Microsystems, Milan, Italy), as described previously [22]. For all specimens a tissue slice was collected at baseline time (T0) and at 24 h intervals for up to 72 h. At each time point, the individual tissues cultures were harvested, formalin-fixed and paraffin-embedded (FFPE) for morphological and immunohistochemical analysis. For 29 samples, tissue was available for the preparation of a second T0 slice that was snap-frozen and utilized for molecular studies. A pathologist (SB) examined the tissue cultures to verify the presence of tumor cells.

### Organotypic tissue cultures and treatment

Tissue slices were cultured as described [22]. For treatment, doxorubicin (10 μM, Sigma-Aldrich, St. Louis, MO, USA) or vehicle (1 μl phosphate-buffered saline (PBS), Gibco Invitrogen, Life Technologies, Carlsbad, CA, USA) was added to the culture medium, and replenished at the same concentration every 24 h.

### Immunohistochemical analysis

All samples were analyzed for morphologic integrity and presence of breast cancer cells by hematoxylin and eosin (H&E) staining. Immunohistochemical scores in tissue cultures and T0 samples were determined in the epithelial tumor cells compartments. Immunostaining was performed for Ki-67 (MIB1, 1:100, Dako, Milan, Italy), estrogen receptor (ER, 1D5, 1:200, Dako), and progesterone receptor (PR, PGR-636, 1:100, Dako) on all samples. Tumor cells immunoreactivity for p53 (Ab5-DO-7, 1:1000, LabVision/NeoMarkers, Fremont, CA, USA), p21^Waf1^ (CP-74, 1:1000, Sigma-Aldrich) and MDM2 (Ab1 IF2, 1:100, Oncogene Science, Wilex Inc., Cambridge, MA, USA) could be assessed on 31 FFPE samples. Analysis of cleaved caspase-3 (Asp175, 5A1E, 1:500, Cell Signaling Technology, Danvers, MA, USA) was carried out on 30 samples. Binding of the individual primary antibody was detected with a secondary antibody of appropriate specificity, and visualized by 4′,6-diamidino-2-phenylindole (DAB) followed by counterstaining with hematoxylin. Negative controls were prepared in the absence of primary antibody and included in each reaction. Three investigators (AF, VV and SB) independently examined and scored all slides. When discrepancies occurred, the cases were reviewed jointly until a consensus was reached. For quantification of proliferative activity, a Ki-67 score was determined at diagnosis as the percentage of positive tumor cells [23]. The data were correlated to the individual responses to doxorubicin in the organotypic cultures. A Ki-67 score in organotypic cultures of breast tumors was calculated as the percentage of positive cells divided by the entire tumor cell population present in the sample. A two-score system for percentage of positive cells and intensity of staining was used to quantify the reactivity for p53, MDM2, p21^waf1^ and cleaved caspase-3. The intensity of staining was expressed in a scale of 0 (absent staining) to 3 (strong staining). A Ki-67/cleaved caspase-3 ratio was expressed as growth index (GI) [24]. Data for each time point were normalized to the samples at baseline (T0). Representative images were obtained using an LMD108 system (Leica Microsystems) and contrast/brightness was adjusted using Photoshop (Adobe Systems Inc., San Jose, CA, USA).

### p53 mutational analysis

DNA was purified from frozen or FFPE samples at T0 and amplified with specific primers for p53 exons 5 to 9 (Table S1 in Additional file 1). Both amplicon strands were sequenced using the BigDye Terminators chemistry (Applied Biosystems, Life Technologies, Carlsbad, CA, USA), and purified using DyeEx 2.0 Spin Kit (Qiagen, Manassas, VA, USA). DNA sequences were performed using a 3130 Genetic Analyzer (Applied Biosystems, Life Technologies).

### RNA purification and reverse transcription

Total RNA was extracted from frozen tissues using RNeasy Mini Kit (Qiagen) according to the supplier’s protocol. One μg of total RNA was reverse-transcribed using High-Capacity cDNA Reverse Transcription Kit (Applied Biosystems, Life Technologies).

### Apoptotic gene profiling

Gene expression analysis of apoptotic-related genes was analyzed in five Responder and five Non Responder cases of organotypic breast tumors using RNA purified from T0 samples before culture by custom Microfluidic Cards technology (Applied Biosystems, Life Technologies). This platform allows for simultaneous expression analysis of 92 apoptosis-related genes (Table S2 in Additional file 1) and three reference genes (ACTB, TBP and HMBS) for target gene relative quantification. Eight hundred ng of cDNA for each sample was loaded in duplicate per card, analyzed using an ABI Prism 7900HT sequence detection system, and targets raw data (Ct values) were converted into relative quantities using GeNorm software [25]. Relative quantity RQ values were then median-normalized and log2-transformed for statistical analysis. All reagents, instruments and software were by Applied Biosystems, Life Technologies.

### Real time RT-PCR (qPCR)

Gene expression levels of tumor necrosis factor-alpha (TNFα), baculoviral IAP repeat- containing 3 (BIRC3), survivin (BIRC5) isoform 1, survivin-2B (isoform 3), or survivin-ΔEx3 (isoform 2) were analyzed in duplicate using gene-specific primers and probes (Table S3 in Additional file 1) and the ABI Prism 7900HT sequence detection system. β-2 microglobulin (β2M) was used as reference gene for target genes relative quantification using the 2^-ΔCt^ formula.

### *In vitro* experiments

Human breast cancer MCF-7, MDA-MB231 and HS578T cells were obtained from the American Type Culture Collection (ATCC, Manassas, VA, USA) and maintained in culture in Dulbecco’s modified Eagle’s medium (DMEM) containing 10% fetal bovine serum (FBS) supplemented with 100 U/ml penicillin/streptomycin and 2 mM L-glutamine at 37°C and 5% CO_2_, as recommended by the supplier. MCF-7 cells (2×10^6^/well in a six-well plate) were transfected with 2 μg of vectors containing survivin-ΔEx3 cDNA or control plasmid [26] in the presence of lipofectamine 2000 (Invitrogen, Life Technologies, Carlsbad, CA, USA) for 24 h. Transfected cells were treated with 1 μM doxorubicin or vehicle (PBS) for 24 h, released for additional 24 h and analyzed for functional experiments.

### Immunofluorescence

Snap-frozen tissue sections derived from seven organotypic breast tumors or MCF-7 cells were fixed in 4% paraformaldehyde (PFA) (Sigma-Aldrich), and incubated with a primary antibody to phospho-histone H2AX (pSer^139^) (γH2AX, 1:700, Sigma-Aldrich) for 16 h at 22°C. Anti-rabbit 647 or 548 Alexa Fluor-conjugated was used as a secondary antibody (Molecular Probes, Invitrogen), and nuclei were stained with 4′,6-diamidino-2-phenylindole (DAPI) (Abbott Laboratories, Abbott Park, IL, USA). Slides were scored by fluorescence microscopy using an AxioImager Z1 microscope (Carl Zeiss, Göttingen, Germany) and photographed images were arranged with Photoshop. For each sample, 100 cells were analyzed and the H2AX phosphorylation index was calculated as a percentage of γH2AX-positive cells [26].

### Cell viability and TUNEL assays

Cell viability was assessed in MCF-7 cells in the presence of 5 mg/ml of 3-(4,5-dimethylthiazol-2-yl)-2,5 diphenyltetrazolium bromide assay (MTT, Sigma-Aldrich) for 4 h at 37°C, and determination of absorbance at 570 nm. Apoptotic cells were quantified by terminal deoxynucleotidyl transferase dUTP nick-end labeling (TUNEL) assay using an ApopTag In Situ Apoptosis Detection Kit (Millipore, Billerica, MA, USA) following the manufacturer’s protocol.

For combination experiments, MCF-7 or HS578T cells were incubated with 5 nM of the survivin small-molecule suppressant YM155 (Selleckchem, Houston, TX, USA) prepared as stock solutions (10 μM) in dimethyl sulfoxide (DMSO) in the presence or absence of doxorubicin (1 to 4 μM, Selleckchem) or vehicle (DMSO). MDA-MB231 cells were incubated with 10 nM YM155. After 24 h incubation, cells under the various conditions tested were analyzed for cell viability by Trypan blue exclusion and light microscopy. Alternatively, treated MCF-7 cells were washed in PBS, pH 7.2, fixed in cold 70% ethanol overnight at 4°C, stained with propidium iodide (0.2 g/ml), and analyzed for DNA content by flow cytometry on a FACSCalibur instrument (Beckman Coulter, Indianapolis, IN, USA). Cell death was quantified in three independent experiments by detection of a population with hypodiploid (sub-G1) DNA content using FlowJo Software (Tree Star, Inc., Ashland, OR, USA). In other experiments, drug-treated MCF-7 cells were analyzed by Western blotting with primary antibodies to cleaved caspase-3 (Asp175) (Cell Signaling), or poly-ADP ribose polymerase (PARP) (46D11) (Cell Signaling). An antibody to β-actin (Sigma-Aldrich) was used as control.

### Synergy studies

MCF-7 cells (1.7×10^4^/cm^2^) were treated simultaneously with YM155 (0.0004 to 0.1 μmol/L) and either doxorubicin, taxol, camptothecin, or etoposide (dose range for each agent 0.004 to 1 μmol/L) in a 7 × 7 matrix of concentrations for 48 h. Cell viability under the various conditions tested was determined by the addition of resazurin to a final concentration of 50 μM and incubation for 5 h. Fluorescence intensity (excitation, 560 nm; emission, 590 nm) was measured on an Envision Xcite Multilabel Reader (Perkin-Elmer, Waltham, MA, USA), and the fractional growth inhibition was determined by normalizing assay wells (n = 2) to the aggregated average responses of positive control (10 μM doxorubicin) and negative control (0.2% DMSO) treatments (n = 12). Synergy between YM155 and chemotherapeutic agents was determined by Bliss independence analyses [27]. The Bliss expectation (E) for a combined response was calculated by the equation: E = (A + B) - (A × B) where A and B are the fractional growth inhibitions of drug A and B at a given dose. The difference between the Bliss expectation and the observed growth inhibition of the combination of drugs A and B at the same dose is the ‘Excess over Bliss’. Excess over Bliss scores = 0 indicates that the combination treatment is additive (as expected for independent pathway effects); Excess over Bliss scores >0 indicates activity greater than additive (synergy); and Excess over Bliss scores <0 indicates the combination is less than additive (antagonism). Each experiment was independently repeated twice with comparable results.

### Statistical analysis

Data were analyzed using Prism 4.0 (GraphPad Inc, La Jolla, CA, USA). Differences among sample groups were analyzed using the unpaired Student’s *t* test. The association between Ki-67 levels and clinicopathological or molecular parameters in the various patients was evaluated by Fisher’s exact test. The expression profiling of apoptotic genes in organotypic breast tumors was analyzed using the web-based BRB-ArrayTools software [28] and Ingenuity Pathway system (Ingenuity Systems Inc., Redwood City, CA, USA). Statistical significance was assumed for a probability value (*P*) less than 0.05.

## Results

### Organotypic breast tumor responses to DNA-damaging agents

**Figure 1:**
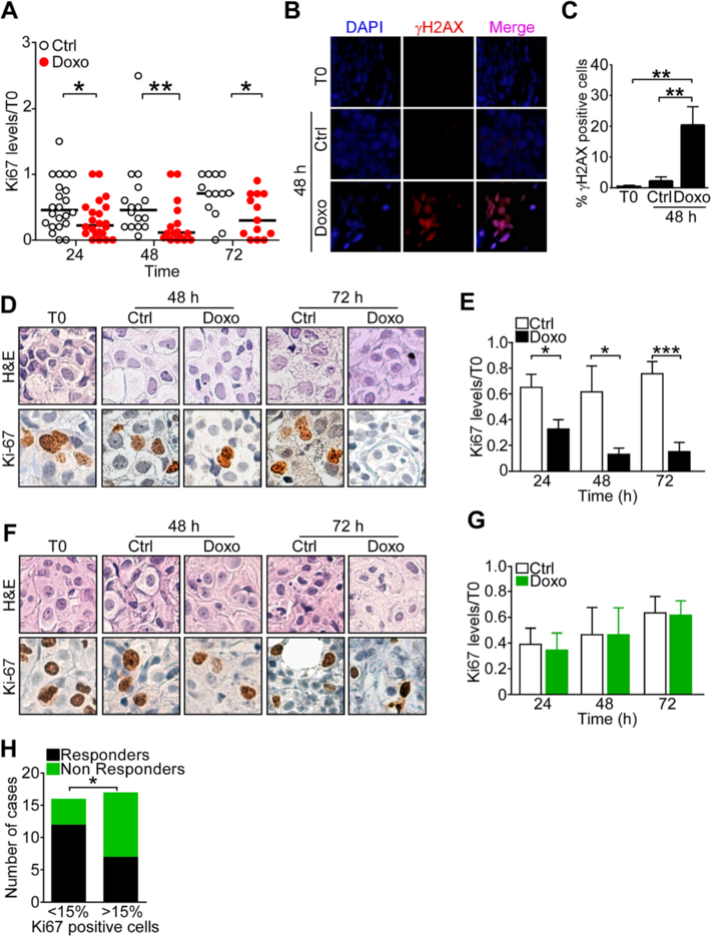
**Response of organotypic breast tumors to doxorubicin. (A) ***Ex vivo* cultures of primary human breast tumors (n = 33) were incubated with 10 μM doxorubicin (Doxo) for the indicated time intervals, and analyzed for Ki-67 expression compared to baseline levels (T0), by immunohistochemistry. *P = 0.03; **P = 0.009 (unpaired *t* test). Each circle corresponds to an individual tumor. **(B, C) ***Ex vivo* tumor samples were analyzed for phosphorylation of histone H2AX (γH2AX) by immunofluorescence, with quantification of γH2AX-reactive cells after 48 h **(C)**. **P = 0.002 (unpaired *t* test). **(D)** Responder breast tumors (n = 19) were treated with Doxo or vehicle (Ctrl) and analyzed for changes in cell proliferation by Ki-67 expression relative to T0 baseline at the indicated time intervals. Original magnification x200. **(E)** Quantification of Ki-67 expression in Responder breast tumors treated with Doxo or Ctrl for the indicated time intervals. *P = 0.02; **P = 0.0003 (unpaired *t* test). **(F)** Non Responder breast tumors (n = 14) were treated with Ctrl or Doxo, and analyzed for Ki-67 expression relative to T0 as in **(D)**. Original magnification x200. **(G)** Quantification of Ki-67 expression in Non Responder breast tumors treated with Doxo or Ctrl for the indicated time intervals. **(H)** Responders and Non Responders breast tumors were analyzed for baseline Ki-67 immunoreactivity (% of positive nuclei). *P = 0.04 (Fisher’s exact test). For all experiments, bars represent mean ± SEM.

We began this study by examining the response of 33 treatment-naïve *ex vivo* human breast tumors [22] to anthracycline-containing therapy. Treatment of organotypic breast tumors with doxorubicin (10 μM) decreased cell proliferation at 24-h time intervals, throughout a 72-h culture (Figure 1A). In contrast, administration of vehicle had no effect on tumor cell proliferation in these settings (Figure 1A). Doxorubicin treatment did not alter the hormone receptor status of *ex vivo* breast tumors, including estrogen and progesterone receptors (Figure S1A and B in Additional file 1). Consistent with a predicted induction of DNA damage, doxorubicin-treated organotypic cultures exhibited high levels of phosphorylated histone H2AX (γH2AX), a cellular marker of double-strand DNA breaks (DSB) (Figure 1B), compared to vehicle-treated cultures or tumors harvested at baseline conditions (Figure 1C). Under these conditions, doxorubicin treatment identified two discrete subgroups of *ex vivo* breast tumors, characterized by radically different proliferative responses. In 19 out of 33 samples (57%), treatment with doxorubicin induced a decrease in proliferating cells over the last time interval (Figure 1D) equal to or greater than 50% (Ki-67 immunoreactivity range, 0 to 50%), compared to control cultures (Figure 1E). These breast tumors were designated as Responders in our study. Conversely, the remaining 14 breast tumors (42%) exhibited no significant decrease in proliferative index throughout exposure to doxorubicin (Figure 1F), quantitatively indistinguishable to that of vehicle-treated cultures (Ki-67 immunoreactivity range 70 to 180%; Figure 1G). These doxorubicin-resistant breast tumors were designated as Non Responders in the study. Consistent with this working classification, organotypic breast tumors in the Responder group exhibited a lower proliferative index at diagnosis with <15% Ki-67-positive cells, whereas tumors in the Non Responder group had higher proliferative index, by Ki-67 staining (mean levels: 16% versus 26%, respectively, *P* = 0.04; Figure 1H). No other significant association was observed between the different levels of Ki-67 immunoreactivity in Responder and Non Responder breast tumors and histologic type (*P* = 0.1), tumor size (*P* = 0.7), lymph node status (*P* = 0.2), hormone receptor expression (ER, *P* = 0.8; PR, *P* = 0.3), or HER2 status (*P* = 0.2). As expected, Ki-67 immunoreactivity displayed a trend to associate with tumor grade (*P* = 0.06).

### Differential regulation of apoptosis in organotypic breast tumors

**Figure 2:**
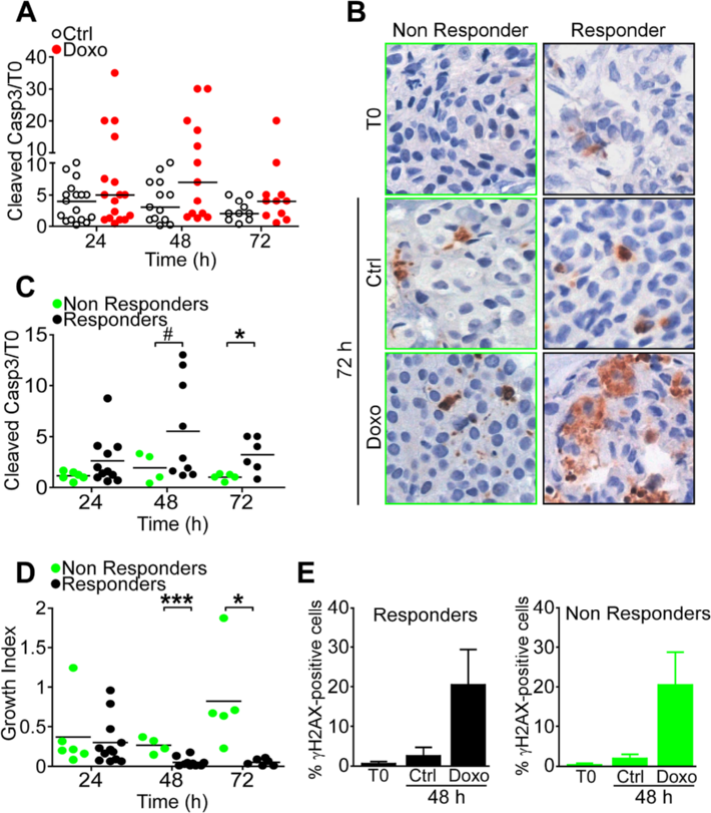
**Differential induction of apoptosis in doxorubicin-treated organotypic breast tumors. (A)** Breast tumors (n = 33) were incubated with 10 μM doxorubicin (Doxo) for the indicated time intervals and analyzed for cleaved caspase-3 (Casp3), by immunohistochemistry. **(B)** Responder (n = 19) versus Non Responder (n = 14) breast tumors were analyzed for Casp3 expression after 72 h treatment with vehicle (Ctrl) or Doxo by immunohistochemistry. Original magnification x 400. **(C)** Quantification of Casp3 immunoreactivity in Non Responder versus Responder breast tumors at the indicated time intervals after Doxo treatment. ^#^*P* = 0.04; *P = 0.02. **(D)** Quantification of growth index (GI) in Responder versus Non Responder breast tumors at the indicated time intervals after Doxo treatment. *P = 0.02; ***P = 0.0005 (unpaired *t* test). Each circle corresponds to an individual tumor. **(E)** γH2AX-reactive cells were quantified after 48 h treatment with Doxo in Responder or Non Responder breast tumors by fluorescence microscopy. For all experiments, bars represent mean ± SEM.

In addition to decreased cell proliferation, treatment of organotypic breast tumors with doxorubicin resulted in increased expression of cleaved, that is active caspase-3, a marker of terminal activation of apoptosis (Figure 2A). When stratified according to the Responder versus Non Responder classification, doxorubicin induced caspase-3 cleavage almost exclusively in the Responder population of breast tumors (Figure 2B and C). In contrast, Non Responder samples had virtually no increase in active caspase-3 generation after doxorubicin treatment throughout a 72-h culture (Figure 2B and C). Similarly, Responder breast tumors exhibited progressive suppression of a GI, calculated as the ratio between Ki-67 immunoreactivity and cleaved caspase-3 in response to doxorubicin treatment (24) (Figure 2D). In contrast, Non Responder tumors continued to exhibit high GI throughout a 72-h doxorubicin treatment (Figure 2D). Despite the extensive differences in proliferative, apoptotic and growth markers (Figures 1, 2), Responder and Non Responder breast tumors showed comparable reactivity for γH2AX induction in response to doxorubicin treatment (Figure 2E).

**Figure 3:**
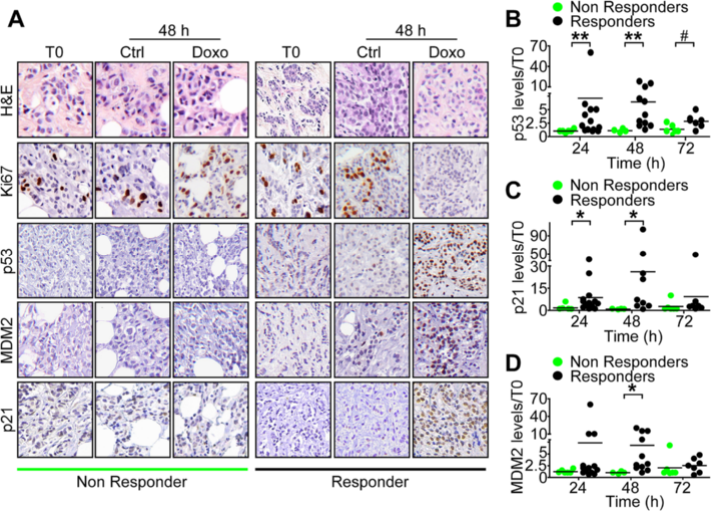
**Differential p53 pathway activation in organotypic breast tumors. (A)** Immunohistochemical analysis of differential expression of p53, p21^Waf1^ or MDM2 in control (Ctrl)- or doxorubicin (Doxo)-treated breast tumors after 48 h. Tissue morphology and proliferative index were determined by hematoxylin and eosin (H&E) and Ki-67 staining of the same cases, respectively. A representative case for Non Responder (left) or Responder (right) breast tumors *ex vivo* is shown. Original magnification, x200. **(B-D)** Quantification of p53 (B), p21^Waf1^ (C) or MDM2 (D) proteins levels in Ctrl or Doxo-treated Responder versus Non Responder breast tumors at the indicated time intervals. ^#^*P* = 0.03 *P = 0.01; **P = 0.007 (unpaired *t* test).

As an upstream regulator of apoptosis and cell cycle arrest in response to DNA damage, we next investigated a potential differential activation of p53 in organotypic breast tumors. Doxorubicin treatment resulted in increased expression of p53 (Figure S2A in Additional file 1), and upregulation of its transcriptional target, p21^waf1^ (Figure S2B in Additional file 1). In contrast, protein levels of the p53 regulator, MDM2 were not significantly affected (Figure S2C in Additional file 1). When analyzed in the functional tumor subgroups, doxorubicin-induced immunoreactivity for p53 (Figure 3A and B) and p21^waf1^ (Figure 3A and C) almost exclusively segregated with Responder tumors throughout a 24- to 48-h treatment. In contrast, Non Responder tumors did not exhibit p53 stabilization (Figure 3A), or upregulation of p21^waf1^ (Figure 3A and C) after exposure to doxorubicin for the same time intervals. Although no changes in MDM2 levels were observed in unfractionated organotypic tumors (Figure S2C in Additional file 1), analysis of tumor subgroups revealed a significant increase in MDM2 protein levels in Responder compared to Non Responder breast tumors (Figure 3A and D). The differences in p53 pathway induction in Responder versus Non Responder breast tumors was independent of p53 gene mutations, as sequence analysis of p53 exons 5 to 9 revealed comparable mutation frequencies among Responder and Non Responder samples (10% and 7%, respectively, *P* = 0.73; Table 1).

### Role of survivin family proteins in doxorubicin resistance in organotypic breast tumors

**Figure 4:**
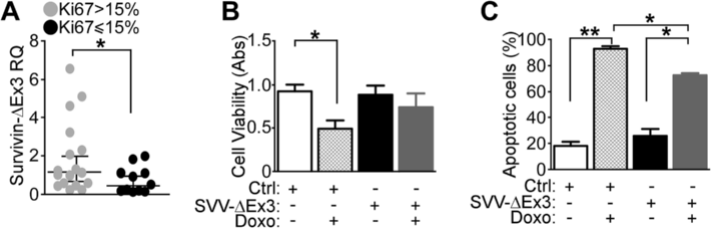
**Role of survivin family proteins in doxorubicin resistance in breast cancer. (A)** Human breast tumors with low (Ki-67 ≤ 15%) or high (Ki-67 > 15%) proliferative index were analyzed for differential expression of survivin-ΔEx3 by qPCR. *P = 0.03. **(B, C)** MCF-7 cells were transfected with survivin-ΔEx3 (SVV-ΔEx3) or control vector (Ctrl), treated with 1 μM doxorubicin or vehicle, and analyzed for cell viability **(B)** or apoptosis **(C)** after 48 h by an 3-(4,5-dimethylthiazol-2-yl)-2,5 diphenyltetrazolium bromide (MTT) or terminal deoxynucleotidyl transferase dUTP nick-end labeling (TUNEL) assay, respectively. *P = 0.01; **P = 0.002 (unpaired *t* test).

To identify a potential molecular basis of doxorubicin resistance in *ex vivo* breast tumors, we next looked at the expression of 92 apoptotic regulatory genes in organotypic breast tumors at T0 baseline. Array profiling studies revealed a global deregulation of p53 pathway activation in Non Responder versus Responder tumors (Figure S3A in Additional file 1), potentially affecting DNA damage-induced cell death, cell cycle transitions, and tumor metabolic reprogramming (Figure S3B in Additional file 1). Specifically, supervised analysis of array profiling data identified BIRC5 (survivin), BIRC3 (cIAP2) and TNFα as the most deregulated transcripts in Non Responder tumors under these conditions (Figure S3A in Additional file 1). Consistent with these findings, wild-type survivin (Figure S3C in Additional file 1), and its alternatively spliced variant survivin-ΔEx3 (*P* = 0.006, Figure S3D in Additional file 1), were validated by quantitative PCR as significantly differentially expressed in Non Responder versus Responder tumors (n = 29). In contrast, the survivin splice variant survivin-2B was comparably expressed in the two subgroups of organotypic breast tumors (Figure S3E in Additional file 1). Despite a trend was observed for differential increased expression of TNFα (Figure S4A in Additional file 1), and BIRC3 (Figure S4B in Additional file 1) in Non Responder compared to Responder tumors, these changes did not reach statistical significance. Although survivin functions as a broad apoptosis inhibitor and mitotic regulator in genetically heterogeneous tumors [29], survivin-ΔEx3 has been selectively associated with modulation of the DNA damage response, selectively in tumors [26]. Accordingly, survivin-ΔEx3 was differentially expressed in breast tumors with high proliferative index, as determined by Ki-67 immunoreactivity (>15% Ki-67^+^ cells; Figure 4A, *P* = 0.03). Mechanistically, overexpression of survivin-ΔEx3 in MCF-7 cells treated with doxorubicin (Figure S5A in Additional file 1) prevented loss of cell viability (*P* = 0.02; Figure 4B), and significantly decreased the number of apoptotic cells (*P* = 0.01; Figure 4C) after doxorubicin treatment. In control experiments, forced expression of survivin-ΔEx3 did not affect DNA foci formation in MCF-7 cells, as determined by γH2AX-nuclear staining (Figure S5B and C in Additional file 1).

### Incorporation of survivin-based molecular therapy in breast cancer treatment

**Figure 5:**
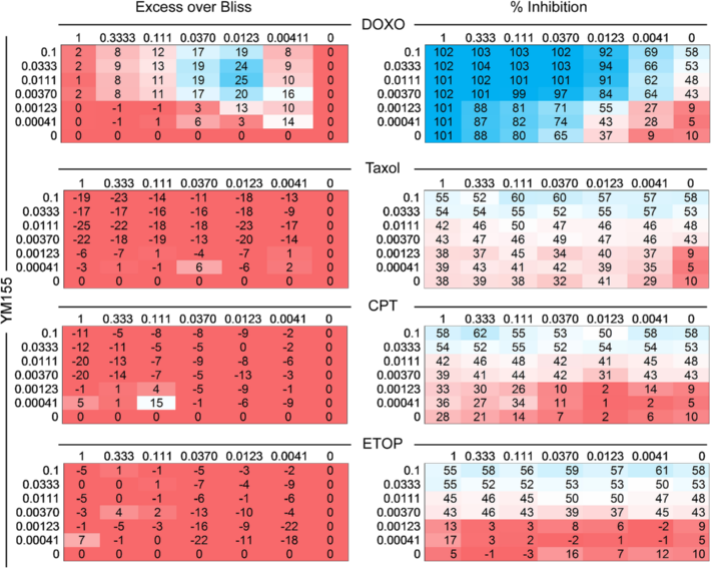
**Combination effects of YM155 and cancer chemotherapeutics on breast cancer cell viability.** MCF-7 cells were treated with YM155 (dose range 0.0004 to 0.100 μmol/L), in combination with either doxorubicin (Doxo), taxol, camptothecin (CPT), or etoposide (ETOP, dose range per each agent, 0.004 to 1 μmol/L) as a 7 × 7 matrix of concentrations in a cell viability assay. The excess over BLISS independence (left) and percentage of growth inhibition (right) are shown as a concentration range for each drug combination.

**Figure 6:**
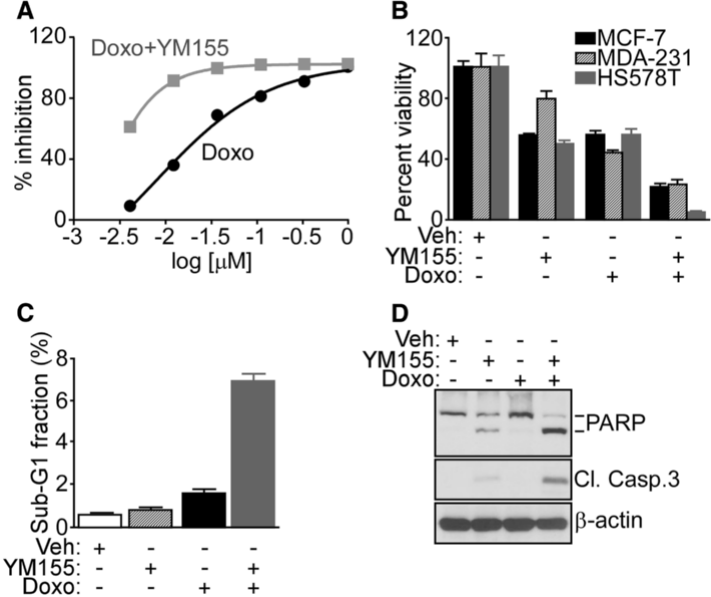
**Therapeutic targeting of survivin pathway in breast cancer cell types. (A)** MCF-7 cells were incubated with the indicated increasing concentrations of doxorubicin (Doxo) alone or in combination with YM155 and analyzed for cell viability after 48 h. Data are representative of one experiment out of two independent determinations. **(B)** The indicated breast cancer cell types were incubated with vehicle (Veh, DMSO), Doxo or YM155, alone or in combination and analyzed for cell viability by Trypan blue exclusion after 48 h. **(C)** MCF-7 cells were treated with the various drug combinations as indicated and analyzed for hypodiploid DNA content (sub-G1), by propidium iodide and flow cytometry. For panels **B** and **C**, data are the mean ± SEM of replicates of a representative experiment out of two independent determinations. **(D)** MCF-7 cells treated as in **B**, **C** were analyzed by Western blotting. The positions of cleaved products for poly-ADP ribose polymerase (PARP) or caspase-3 are indicated.

The results above suggest that survivin family proteins may function as key determinants of doxorubicin-resistance in organotypic breast tumors. To further test this possibility, we next used a high-throughput format to examine combination regimens of first-line anthracycline chemotherapy plus the small-molecule survivin suppressant, sepantronium bromide (YM155) currently evaluated in the clinic [30]. The combination of YM155 plus doxorubicin in these settings produced extensive antitumor synergy against breast cancer MCF-7 cells (Figure 5), and robust inhibition of cell proliferation at various concentrations of the two agents tested (Figure 6A). In contrast, the combination of YM155 with taxol, camptothecin (CPT) or etoposide was not synergistic and produced suboptimal antitumor activity against breast cancer cells (Figure 5). Consistent with these findings, the addition of YM155 significantly enhanced doxorubin-mediated killing of a panel of genetically heterogeneous breast cancer cell types, compared to each treatment alone (Figure 6B). When analyzed for mechanisms of antitumor activity, the combination of YM155 plus doxorubicin increased the fraction of tumor cells with hypodiploid, that is sub-G1 DNA content by flow cytometry (Figure 6C), with increased cleavage of canonical apoptosis markers, including PARP and caspase-3, by Western blotting (Figure 6D).

## Discussion

In this study, we have shown that fresh, treatment-naïve organotypic human breast tumors closely recapitulating the human disease [22] can be stratified into two discrete subgroups that differ sharply in their response to doxorubicin with respect to proliferative status, p53 function and modulation of apoptosis. Doxorubicin-insensitive (that is Non Responder) tumors selectively contained high levels of survivin family proteins, including the alternatively spliced survivin-ΔEx-3 isoform, which was directly implicated in treatment resistance. Conversely, high-throughput targeting this pathway with a small-molecule survivin suppressant currently in the clinic (YM155) selectively synergized with doxorubicin, and restored apoptosis in heterogeneous breast cancer cell types.

Despite the advent of molecular therapies [2], and the improved survival of patients with advanced disease [31], the emergence of treatment-resistant breast cancer, in particular to first-line anthracycline-containing chemotherapy, remains a formidable challenge [10], with limited therapeutic options. The organotypic approach described here [22] identified a functional subset of treatment-naïve breast tumors with nearly complete insensitivity to doxorubicin, characterized by no significant reduction in cell proliferation or appearance of apoptosis in response to treatment. At least in the patient series examined here, the Non Responder phenotype did not appear to segregate with other markers of poor outcome in breast cancer, for instance HER2 overexpression/amplification or p53 mutations. Although analysis of a larger patient cohort may be required to conclusively address this point, the high proliferative status constitutively observed in Non responder breast tumors by Ki-67 staining [32], has been previously associated with drug-resistant disease and poor outcome [33].

The mechanisms of primary drug resistance in breast cancer are still largely elusive [34], but there is evidence that aberrant overexpression of antiapoptotic molecules, in particular survivin [29], confers insensitivity to molecular [35,36] or endocrine [37] therapies, resulting in shortened overall survival [38]. Here, the increased expression of survivin and its alternatively spliced survivin-ΔEx3 variant provided for the single, most significant deregulation of apoptotic pathways in Non Responder breast tumors, and recombinant expression of survivin-ΔEx3 was sufficient, alone, to confer doxorubicin resistance in model breast cancer cell types. It is possible that deregulation of the survivin pathway in Non Responder tumors may reflect the defective p53 responses that were also observed in these patients, as loss of p53-dependent repression of the survivin promoter [39] has been associated with increased survivin gene transcription, including its alternatively spliced variants [40].

Consistent with the data presented here, survivin-ΔEx3 has been recognized as a *bona fide* mediator of cytoprotection, elevating an antiapoptotic threshold in tumors [41]. However, there is also evidence that this molecule may function as a sensor of DNA damage, specifically DSB, and selectively in the transformed cell population [26]. A mechanistic underpinning of this response has been proposed, involving isoform-specific phosphorylation of survivin-ΔEx3 by the checkpoint kinase, Chk2, and time-dependent recruitment of γH2AX, a marker of unrepaired DNA damage [42], to nuclear foci [26]. This scenario may be relevant to the data presented here, as increased expression of survivin-ΔEx3 in Non Responder tumors may promote early recruitment of the DNA repair machinery to DSB, and together with a higher antiapoptotic threshold, limit the anticancer activity of genotoxic stress, including doxorubicin [26]. Indeed survivin-ΔEx3 overexpression may counter the preferential killing of highly proliferating tumor cells by doxorubicin, thus diminishing the efficacy of chemotherapy against this potentially susceptible cellular compartment.

Accordingly, retrospective bioinformatics analyses have linked high expression of survivin-ΔEx3 to unfavorable outcome in various patient series [26], further supporting the more general role of survivin as a poor prognostic marker in breast cancer [43].

Although there is overwhelming evidence that survivin is an important therapeutic target in disparate tumors, including breast cancer [44], the portfolio of survivin antagonists suitable to target this pathway in the clinic has remained disappointingly narrow [15]. Currently, only the small-molecule sepantronium bromide, YM155 [45], proposed to act as a transcriptional suppressant of the survivin locus, has shown good tolerability and preliminary evidence of activity in early-phase trials [44]. On the other hand, survivin is overexpressed in virtually every human tumor [15], making it difficult to identify discrete patient subset(s) likely to benefit from YM155-based therapy, and only limited information is available to rationally guide the incorporation of survivin antagonist(s) in effective combination regimens. The present study may help bridge this gap, uncovering a synergistic response between YM155 and doxorubicin that induced apoptosis in heterogeneous breast cancer cell types. Consistent with a role of survivin family proteins in DNA repair mechanisms [26], YM155 triggered a DNA damage response in tumor cells [46], and potentiated the antitumor activity of ionizing radiation in non-small cell lung cancer [47]. Conversely, the combination of YM155 plus taxol, etoposide or camptothecin was not synergistic in our high-throughput analysis, and had minimal anticancer activity in breast cancer cells. This is reminiscent of other data in the clinic, where the combination of YM155 plus carboplatin/paclitaxel produced only limited responses in non-small cell lung cancer patients [48].

## Conclusions

In summary, we used fresh cultures of treatment-naïve organotypic breast tumors that closely mimic the disease in patients [22] to identify survivin family proteins as drivers of primary doxorubicin resistance across breast cancer subgroups. Despite the introduction of YM155 in the clinic over five years ago [30], the suitability of this treatment in breast cancer has not been clearly demonstrated. The results presented here suggest that incorporation of YM155 in anthracycline-containing chemotherapy may result in greater clinical activity across heterogeneous breast cancer subtypes, and potentially overcome constitutive treatment resistance in these patients.

## Abbreviations

Casp3: cleaved caspase-3; CPT: camptothecin; Ctrl: control/vehicle; DAB: 3,3*'*-diaminobenzidine; DAPI: 4*'*,6-diamidino-2-phenylindole; DLC: ductal and lobular carcinoma; DMEM: Dulbecco’s modified Eagle’s medium; DMSO: dimethyl sulfoxide; Doxo: doxorubicin; DSB: double-strand DNA breaks; ER: estrogen eceptor; ETOP: etoposide; FBS: fetal bovine serum; FFPE: formalin-fixed and paraffin-embedded; GI: growth index; H&E: hematoxylin and eosin; HER2: epidermal growth factor receptor-2; IAP: inhibitor of apoptosis; IDC: invasive ductal carcinoma; ILC: invasive lobular carcinoma; mTOR: mammalian target of rapamycin; MTT: 3-(4,5-dimethylthiazol-2-yl)-2,5 diphenyltetrazolium bromide; PARP: poly-ADP ribose polymerase; PBS: phosphate-buffered saline; PC: papillary carcinoma; PFA: paraformaldehyde; PI3K: phosphatidylinositol 3-kinase; PR: progesterone receptor; RQ: relative quantity; SVV-ΔEx3: survivin-ΔEx3; T0: baseline time; TNFα: tumor necrosis factor-alpha; TUNEL: terminal deoxynucleotidyl transferase dUTP nick-end labeling; VEGF: vascular endothelial cell growth factor; Veh: vehicle-DMSO; γH2AX: phosphorylated histone H2AX.

## Competing interests

The authors disclose no potential conflicts of interests.

## Authors’ contributions

AF and VV designed and performed experiments and analyzed data. GPM provided patients’ clinical information. DT carried out the immunohistochemical experiments. AL performed transfection experiments. DCS and DR carried out combination experiments and synergy studies. DCA and SB conceived of the study, designed and directed the experiments. AF, VV, DCA and SB wrote the manuscript. All authors read and approved the final manuscript.

## Supplementary Material

Additional file 1: Table S1 p53 primers used in this study. **Table S2.** The 92 apoptosis-related genes analyzed in Responder and Non Responder organotypic breast tumors. **Table S3.** Primers and probes used for quantification of survivin isoforms. **Figure S1.** Effect of genotoxic stress on hormone receptor expression in organotypic breast tumors. **Figure S2.** Modulation of p53-dependent responses in organotypic breast tumors. **Figure S3.** Differential expression of survivin family proteins in organotypic breast tumors. **Figure S4.** Expression of apoptosis regulators in organotypic breast tumors. **Figure S5.** Effect of Survivin-ΔEx3 on the DNA-damage response. Click here for file
