# Supplementary material for: Survivin family proteins as novel molecular determinants of doxorubicin resistance in organotypic human breast tumors
Source: Breast Cancer Res. 2014 May 30;16(3):R55. doi: 10.1186/bcr3666 (PMC4076638; doi:10.1186/bcr3666)
Supplement: Additional file 1: Table S1 — p53 primers used in this study. Table S2. The 92 apoptosis-related genes analyzed in Responder and Non Responder organotypic breast tumors. Table S3. Primers and probes used for quantification of survivin isoforms. Figure S1. Effect of genotoxic stress on hormone receptor expression in organotypic breast tumors. Figure S2. Modulation of p53-dependent responses in organotypic breast tumors. Figure S3. Differential expression of survivin family proteins in organotypic breast tumors. Figure S4. Expression of apoptosis regulators in organotypic breast tumors. Figure S5. Effect of Survivin-ΔEx3 on the DNA-damage response. [file bcr3666-S1.doc]

**Ms.9774005031155445_REV1**

**SURVIVIN FAMILY PROTEINS AS NOVEL MOLECULAR DETERMINANTS OF DOXORUBICIN RESISTANCE IN ORGANOTYPIC HUMAN BREAST TUMORS**

Alice Faversani, Valentina Vaira, Giacomina P. Moro, Delfina Tosi, Alessia Lopergolo,

David C. Schultz, Dayana Rivadeneira, Dario C. Altieri and Silvano Bosari

**SUPPLEMENTAL MATERIAL**

Supplemental Tables 1-3

Supplemental Figures 1-5

**Table S1. p53 primers used in this study.** The sequences of the primers used to amplify and sequence p53 exons 5 to 9 are indicated.

| **Exon** | **Forward (5'-3')** | **Reverse (5'-3')** |
| --- | --- | --- |
| 5 | TCAGTGAGGAATCAGAGGCC | CTGACTTTCAACTCTGTCTCC |
| 6 | CTGGAGAGACGACAGGGCTG | CCAGAGACCCCAGTTGCAAAC |
| 7 | AAGGCGCACTGGCCTCATCTT | TCAGCGGCAAGCAGAGGCTG |
| 8 | GGACAGGTAGGACCTGATTTCCTTA | TGCACCCTTGGTCTCCTCCAC |
| 9 | GGTGGAGGAGACCAAGGGTGCAGTT | CTGGAAACTTTCCACTTGAT |

**Table S2**. The ninety-two apoptosis-related genes analyzed in Responder and Non Responder organotypic breast tumors are listed in alphabetical order. The genes ID (from Entrez^a^), and a brief description of the genes function^b^ are provided.

| **Gene ID[[1]](#footnote-2)** | **Gene Symbol** | **Gene Name** | **Gene description and function[[2]](#footnote-3)** |
| --- | --- | --- | --- |
| 26574 | AATF | Apoptosis-Antagonizing Transcription Factor | Inhibitor of the histone deacetylase HDAC1, leading to activation of E2F target genes and cell cycle progression |
| 22985 | ACIN1 | Apoptotic Chromatin Condensation Inducer 1 | It induces apoptotic chromatin condensation after activation by CASP3 |
| 317 | APAF1 | Apoptotic Peptidase Activating Factor 1 | It initiates apoptosis by binding and cleaving Caspase 9 after Cytochrome C binding |
| 472 | ATM | Ataxia Telangiectasia Mutated | It is a cell cycle kinase chekpoint important for DNA damage response and for genome stability |
| 572 | BAD | BCL2-associated agonist of cell death | Member of BCL2 protein family with pro-apoptotic activity |
| 581 | BAX | BCL2-associated X protein1 | Member of BCL2 protein family with pro-apoptotic activity. It interacts with and antagonizes BCL2 |
| 8915 | BCL10 | B-cell CLL/lymphoma 10 | It induces apoptosis and activates NFkB |
| 596 | BCL2 | B-cell CLL/lymphoma 2 | Its an outer mithocondrial membrane protein which inhibits apoptosis |
| 597 | BCL2A1 | BCL2-related protein A1 | Member of BCL2 protein family with anti-apoptotic activity. It is able to block Cytochrome C release and caspase activation |
| 598 | BCL2L1 | BCL2-like 1 | Member of BCL2 protein family which regulates cell death by blocking the mitocondrial membrane channel (VDAC) |
| 10018 | BCL2L11 | BCL2-like 11 (apoptosis facilitator) | Member of BCL2 protein family which interacts with other BCL2 family members and activates cell death |
| 599 | BCL2L2 | BCL2-like 2 | Member of BCL2 protein family which inhibits apoptosis |
| 51283 | BFAR | bifunctional apoptosis regulator | Apoptosis regulator. Has anti-apoptotic activity, both for apoptosis triggered via death-receptors and via mitochondrial factors |
| 637 | BID | BH3 interacting domain death agonist | Member of BCL2 protein family which inhibits apoptosis by interacting with Bax or inhibiting BCL2 |
| 638 | BIK | BCL2-interacting killer (apoptosis-inducing) | Pro-apoptotic protein target of anti-apoptotic proteins |
| 4671 | BIRC1 | NLR family, apoptosis inhibitory protein | Anti-apoptotic protein |
| 329 | BIRC2 | Baculoviral IAP repeat containing 2 | It inhibits apoptosis by interacting with tumor necrosis factor receptor-associated factors TRAF1 and TRAF2 |
| 330 | BIRC3 | Baculoviral IAP repeat containing 3 | It inhibits apoptosis by interacting with tumor necrosis factor receptor-associated factors TRAF1 and TRAF3 |
| 332 | BIRC5 (SVV) | Baculoviral IAP repeat containing 5 | Member of IAP proteins family which inhibits anti-apoptotic proteins |
| 57448 | BIRC6 | Baculoviral IAP repeat containing 6 | This protein inhibits apoptosis by facilitating the degradation of apoptotic proteins by ubiquitination. |
| 79444 | BIRC7 | Baculoviral IAP repeat containing 7 | Member of IAP proteins family and inhibitor of apoptosis |
| 666 | BOK | BCL2-related ovarian killer | It is a pro-apoptotic BCL-2 protein identified in the ovary |
| 10392 | CARD4 | Nucleotide-binding oligomerization domain containing 1 | It is a cytosolic protein which anhances caspase-9 mediated apoptosis |
| 843 | CASP10 | Caspase 10, apoptosis-related cysteine peptidase | It is a member of Caspase family. It cleaves and activates caspases 3 and 7, and it is processed by caspase 8 |
| 835 | CASP2 | Caspase 2, apoptosis-related cysteine peptidase | It is a member of Caspase family |
| 836 | CASP3 | Caspase 3, apoptosis-related cysteine peptidase | It is a member of Caspase family. It cleaves and activates caspases 6, 7 and 9 and it is processed by caspase 8, 9 and 10 |
| 839 | CASP6 | Caspase 6, apoptosis-related cysteine peptidase | It is a member of Caspase family. It is processed by caspase 7, 8 and 10 and it is considered as a downstream enzyme in the caspase activation cascade |
| 840 | CASP7 | Caspase 7, apoptosis-related cysteine peptidase | It is a member of Caspase family. Its precursor is processed by caspase 3 and 10 |
| 841 | CASP8 | Caspase 8, apoptosis-related cysteine peptidase | It is a member of Caspase family and it is involved in cell death induced by Fas |
| 9994 | CASP8AP2 | Caspase 8 associated protein 2 | It interacts with the death-effector domain of caspase 8 and it plays a regolatory role in Fas-mediated apoptosis |
| 842 | CASP9 | Caspase 9, apoptosis-related cysteine peptidase | It is a member of Caspase family and it is activated by APAF1. When it is activated, it cleaves Caspase 3 |
| 958 | CD40 | CD40 molecule | Member of the TNF-receptor superfamily, it is very important in a variety of immune and inflammatory responses |
| 959 | CD40LG | CD40 ligand | It is a protein expressed on T cells surface. It regulates B cells function by engaging CD40 on B cell surface |
| 1026 | CDKN1A | Cyclin-dependent kinase inhibitor 1A | It binds to and inhibits the activity of cyclin-CDK2 or -CDK4 complexes, and functions as a regulator of cell cycle progression at G1 |
| 1027 | CDKN1B | Cyclin-dependent kinase inhibitor 1B | It is a cycline dependent kinase inhibitor with similariy with CDKN1A |
| 1029 | CDKN2A | Cyclin-dependent kinase inhibitor 2A | It is a cycline dependent kinase inhibitor and functions as an inhibitor of CDK4 |
| 8837 | CFLAR | CASP8 and FADD-like apoptosis regulator | It is a regulator of apoptosis structurally similar to Caspase 8 |
| 1111 | CHEK1 | CHK1 checkpoint homolog | It is required for checkpoint mediated cell cycle arrest in response to DNA damage or the presence of unreplicated DNA |
| 11200 | CHEK2 | CHK2 checkpoint homolog | It is a cell cycle checkpoint regulator and putative tumor suppressor. |
| 8738 | CRADD | CASP2 and RIPK1 domain containing adaptor with death domain | The protein encoded by this gene is a death domain (CARD/DD)-containing protein and has been shown to induce cell apoptosis |
| 1434 | CSE1L | CSE1 chromosome segregation 1-like | It plays a role in apoptosis and cell proliferation |
| 1603 | DAD1 | Defender against cell death 1 | Negative regulator of programmed cell death |
| 23604 | DAPK2 | Death-associated protein kinase 2 | Positive regulator of apoptosis |
| 1616 | DAXX | Death-domain associated protein | Multifunctional protein with cytoplasmic and nuclear localization. It is believed that it regulates apoptosis |
| 1676 | DFFA | DNA fragmentation factor | It is substrate of Caspase-3 and triggers DNA fragmentation during apoptosis |
| 56616 | DIABLO | Diablo, IAP-binding mitochondrial protein | It is an inhibitor of apoptosis protein (IAP)-binding protein. During apoptosis, it enters in the cytosol and moderates the caspase inhibition of IAPs |
| 1981 | EIF4G1 | Eukaryotic translation initiation factor 4 gamma, 1 | Component of the protein complex eIF4F, which is involved in the recognition of the mRNA cap, ATP-dependent unwinding of 5'-terminal secondary structure and recruitment of mRNA to the ribosome |
| 8772 | FADD | Fas (TNFRSF6)-associated via death domain | Adaptor molecule that interacts with various cell surface receptors and mediates cell apoptotic signals |
| 355 | FAS | Fas (TNF receptor superfamily, member 6) | Member of the TNF-receptor superfamily that plays a central role in the physiological regulation of programmed cell death |
| 356 | FASLG | Fas ligand (TNF superfamily, member 6) | Ligand of Fas. The interaction between Fas and its igand is critical in triggering apoptosis of some types of cells such as lymphocytes |
| 1647 | GADD45A | Growth arrest and DNA-damage-inducible, alpha | It responds to DNA damage agents. It stimulates DNA excision repair in vitro and inhibits entry of cells into S phase |
| 3308 | HSPA4 | Heat shock 70kDa protein 4 | Member of the heat shock proteins family |
| 4049 | LTA | Lymphotoxin alpha | It is a cytokine and member of the tumor necrosis factor family. It is secreted by lymphocytes and plays a role in apoptosis |
| 4050 | LTB | Lymphotoxin beta | It is a type II membrane protein of the TNF family. It anchors lymphotoxin-alpha to the cell surface through heterotrimer formation |
| 4055 | LTBR | Lymphotoxin beta receptor | It is a member of the tumor necrosis factor family of receptors. It is expressed on the surface of most cells and its activation can trigger apoptosis |
| 4170 | MCL1 | Myeloid cell leukemia sequence 1 | Anti-apoptotic protein and member of BCL2 protein family |
| 4193 | MDM2 | Mdm2 p53 binding protein homolog | It is a nuclear phosphoprotein that binds and inhibits transactivation by tumor protein p53, as part of an autoregulatory negative feedback loop |
| 4194 | MDM4 | Mdm4 p53 binding protein homolog | It inhibits p53/TP53- and TP73/p73-mediated cell cycle arrest and apoptosis by binding its transcriptional activation domain |
| 4615 | MYD88 | Myeloid differentiation primary response gene (88) | Cytosolic adapter protein that plays a central role in the innate and adaptive immune response |
| 10401 | PIAS3 | Protein inhibitor of activated STAT, 3 | It functions as a SUMO-E3 ligase which catalyzes the covalent attachment of a SUMO protein to specific target substrates |
| 29108 | PYCARD | PYD and CARD domain containing | Its protein domain PYD and CARD mediate assembly of large signaling complexes in the inflammatory and apoptotic signaling pathways via the activation of caspase |
| 8737 | RIPK1 | Receptor (TNFRSF)-interacting serine-threonine kinase 1 | Essential adapter molecule for the activation of NF-kappa-B |
| 8767 | RIPK2 | Receptor-interacting serine-threonine kinase 2 | It contains a C-terminal caspase activation and recruitment domain (CARD), and is a component of signaling complexes in both the innate and adaptive immune pathways |
| 6772 | STAT1 | Signal transducer and activator of transcription 1 | This protein mediates the expression of a variety of genes, which is thought to be important for cell viability in response to different cell stimuli and pathogens |
| 6774 | STAT3 | Signal transducer and activator of transcription 3 | Transcription factor that binds to the interleukin-6 (IL-6)-responsive elements identified in the promoters of various acute-phase protein genes |
| 10010 | TANK | TRAF family member-associated NFKB activator | Member of the TRAF (tumor necrosis factor receptor-associated factor) family of proteins that associate with and transduce signals from members of the tumor necrosis factor receptor superfamily |
| 7124 | TNF | Tumor necrosis factor | Cytokine that is mainly secreted by macrophages and is involved in the regulation of a wide spectrum of biological processes including cell proliferation, differentiation, apoptosis, lipid metabolism, and coagulation |
| 8797 | TNFRSF10A | Tumor necrosis factor receptor superfamily, member 10a | Member of the TNF-receptor superfamily. This receptor is activated by tumor necrosis factor-related apoptosis inducing ligand (TNFSF10/TRAIL), and thus transduces cell death signal and induces cell apoptosis |
| 8795 | TNFRSF10B | Tumor necrosis factor receptor superfamily, member 10d | Member of the TNF-receptor superfamily. This receptor can be activated by tumor necrosis factor-related apoptosis inducing ligand (TNFSF10/TRAIL/APO-2L), and transduces an apoptosis signal |
| 8793 | TNFRSF10D | Tumor necrosis factor receptor superfamily, member 10b | Member of the TNF-receptor superfamily. It has been shown to play an inhibitory role in TRAIL-induced cell apoptosis |
| 8764 | TNFRSF14 | Tumor necrosis factor receptor superfamily, member 14 | Member of the TNF-receptor superfamily. Its cytoplasmic region was found to bind to several TRAF family members, which may mediate the signal transduction pathways that activate the immune response. |
| 7132 | TNFRSF1A | Tumor necrosis factor receptor superfamily, member 1A | It is a member of the TNF-receptor superfamily and is one of the major receptors for the tumor necrosis factor-alpha |
| 7133 | TNFRSF1B | Tumor necrosis factor receptor superfamily, member 1B | It is a member of the TNF-receptor superfamily. This protein and TNF-receptor 1 form a heterocomplex that mediates the recruitment of two anti-apoptotic proteins, c-IAP1 and c-IAP2 |
| 7293 | TNFRSF4 | Tumor necrosis factor receptor superfamily, member 4 | It is a member of the TNF-receptor superfamily. This receptor has been shown to activate NF-kappaB through its interaction with adaptor proteins TRAF2 and TRAF5 |
| 939 | TNFRSF7 | Tumor necrosis factor receptor superfamily, member 7 | It is a member of the TNF-receptor superfamily |
| 943 | TNFRSF8 | Tumor necrosis factor receptor superfamily, member 8 | It is a member of the TNF-receptor superfamily. It is a positive regulator of apoptosis |
| 3604 | TNFRSF9 | Tumor necrosis factor receptor superfamily, member 9 | It is a member of the TNF-receptor superfamily. It contributes to the clonal expansion, survival, and development of T cells |
| 8743 | TNFSF10 | Tumor necrosis factor (ligand) superfamily, member 10 | It is a cytokine that belongs to the tumor necrosis factor (TNF) ligand family which preferentially induces apoptosis in transformed and tumor cells |
| 8600 | TNFSF11 | Tumor necrosis factor (ligand) superfamily, member 11 | Member of the tumor necrosis factor (TNF) cytokine family and ligand of the TNFRSF14 |
| 8740 | TNFSF14 | Tumor necrosis factor (ligand) superfamily, member 14 | Member of the tumor necrosis factor (TNF) cytokine family which activates antiapoptotic kinase AKT/PKB through a signaling complex involving SRC kinase and TRAF7 |
| 7292 | TNFSF4 | Tumor necrosis factor (ligand) superfamily, member 4 | Member of the tumor necrosis factor (TNF) cytokine family. This cytokine is a ligand for receptor TNFRSF4/OX4 |
| 970 | TNFSF7 | Tumor necrosis factor (ligand) superfamily, member 7 | Member of the tumor necrosis factor (TNF) cytokine family. |
| 944 | TNFSF8 | Tumor necrosis factor (ligand) superfamily, member 8 | Member of the tumor necrosis factor (TNF) cytokine family. It is a ligand for TNFRSF8/CD30, which is a cell surface antigen and a marker for Hodgkin lymphoma and related hematologic malignancies. |
| 8744 | TNFSF9 | Tumor necrosis factor (ligand) superfamily, member 9 | Member of the tumor necrosis factor (TNF) cytokine family. It is a bidirectional signal transducer that acts as a ligand for TNFRSF9/4-1BB, which is a costimulatory receptor molecule in T lymphocytes. |
| 7157 | TP53 | Tumor protein p53 | It responds to diverse cellular stresses to regulate target genes that induce cell cycle arrest, apoptosis, senescence, DNA repair, or changes in metabolism |
| 7185 | TRAF1 | TNF receptor-associated factor 1 | It is a member of the TNF receptor (TNFR) associated factor (TRAF) protein family. It forms a heterodimeric complex with TRAF2, which is required for TNF-alpha-mediated activation of MAPK8/JNK and NF-kappaB |
| 7186 | TRAF2 | TNF receptor-associated factor 2 | It is a member of the TNF receptor (TNFR) associated factor (TRAF) protein family. It forms a heterodimeric complex with TRAF1 |
| 7187 | TRAF3 | TNF receptor-associated factor 3 | It is a member of the TNF receptor (TNFR) associated factor (TRAF) protein family. This protein participates in the signal transduction of CD40, a TNFR family member important for the activation of the immune response |
| 9618 | TRAF4 | TNF receptor-associated factor 4 | It is a member of the TNF receptor (TNFR) associated factor (TRAF) protein family |
| 7188 | TRAF5 | TNF receptor-associated factor 5 | It is a member of the TNF receptor (TNFR) associated factor (TRAF) protein family |
| 7189 | TRAF6 | TNF receptor-associated factor 6 | It is a member of the TNF receptor (TNFR) associated factor (TRAF) protein family. It mediates the signaling also from the members of the Toll/IL-1 family |
| 10293 | TRAIP | TRAF interacting protein | It binds TRAF1 and TRAF2 and is part of the receptor-TRAF signaling complex |

**Table S3. Primers and probes used for quantification of survivin isoforms.**

| **Gene ID** | **Isoform** | **Forward primer 5'-3'** | **Reverse primer 5'-3'** | **Probe 5'-3'** |
| --- | --- | --- | --- | --- |

| SVV-WT | Isoform 1 | Hs00153353_m1 assay[[3]](#footnote-4) | | |
| --- | --- | --- | --- | --- |
| SVV-ΔEX3 | Isoform 2 | TGGAAGGCTGGGAGCCA | TCGCAGTTTCCTCAAATTCTTTCT | 6-FAM-CGACCCCATGCAAAGGAAACCAAC-TAMRA |
| SVV-2b | Isoform 3 | TTCAAGGAGCTGGAAGGCTG | CAAAGTGCTGGTATTACAGGCGT | 6-FAM-TTGGGCCGGGCACGGTG-TAMRA |

**SUPPLEMENTAL FIGURES and LEGENDS**

**
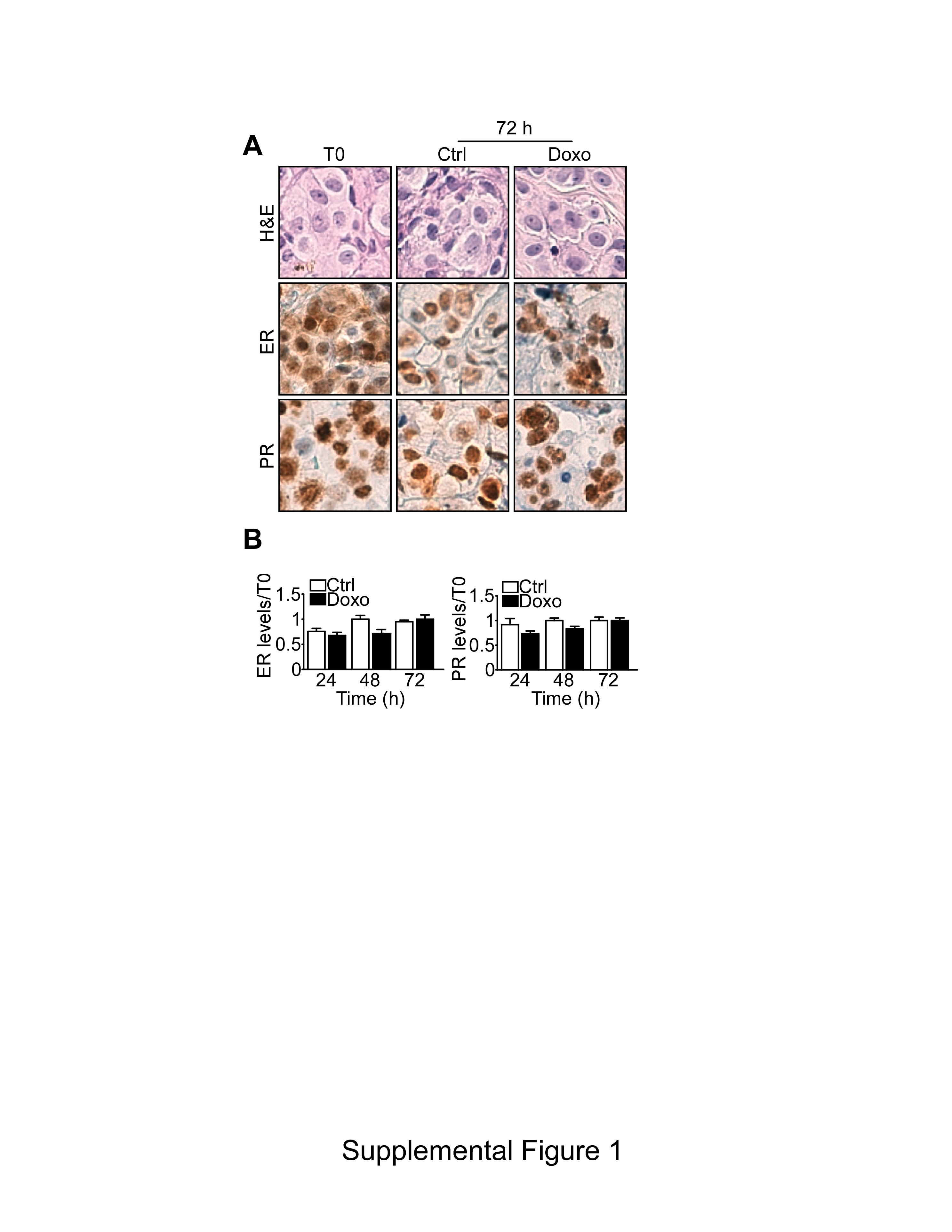
**

**Figure S1.** Effect of genotoxic stress on hormone receptor expression in organotypic breast tumors. A, Precise-thick organotypic tissue cultures from thirty-three breast cancers were maintained in culture for up to 72 h with (Doxo) or without (Ctrl) doxorubicin. Cultures were harvested every 24 h and estrogen (ER) or progesterone (PR) receptor expression was assessed by immunohistochemistry. B, Quantification of ER or PR as detected in Responder (n=19) or Non Responder (n=14) breast tumors at the indicated time intervals. Bars, mean±SEM.

**
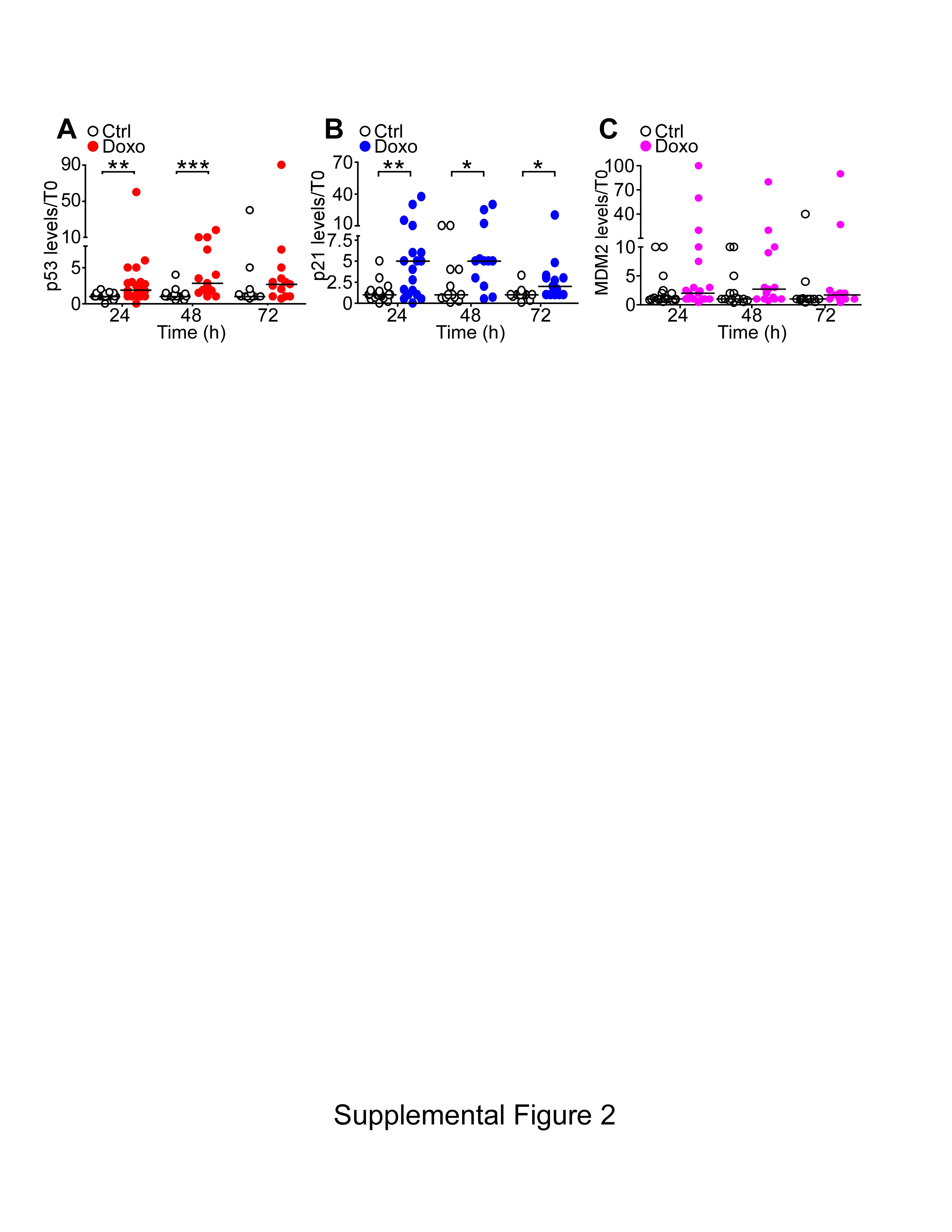
**

**Figure S2.** Modulation of p53-dependent responses in organotypic breast tumors. The expression levels of p53 (A), p21^Waf1^ (B), or MDM2 (C) were quantified by immunohistochemistry in organotypic breast tumors treated with vehicle (Control) or doxorubicin (Doxo) for the indicated times. Proteins levels in the various cultures were quantified relative to untreated samples (T0), and expressed as fold difference. Each symbol corresponds to an individual tumor sample. *, *P*=0.01; **, *P*=0.004; ***, *P*=0.0006 (unpaired *t* test).


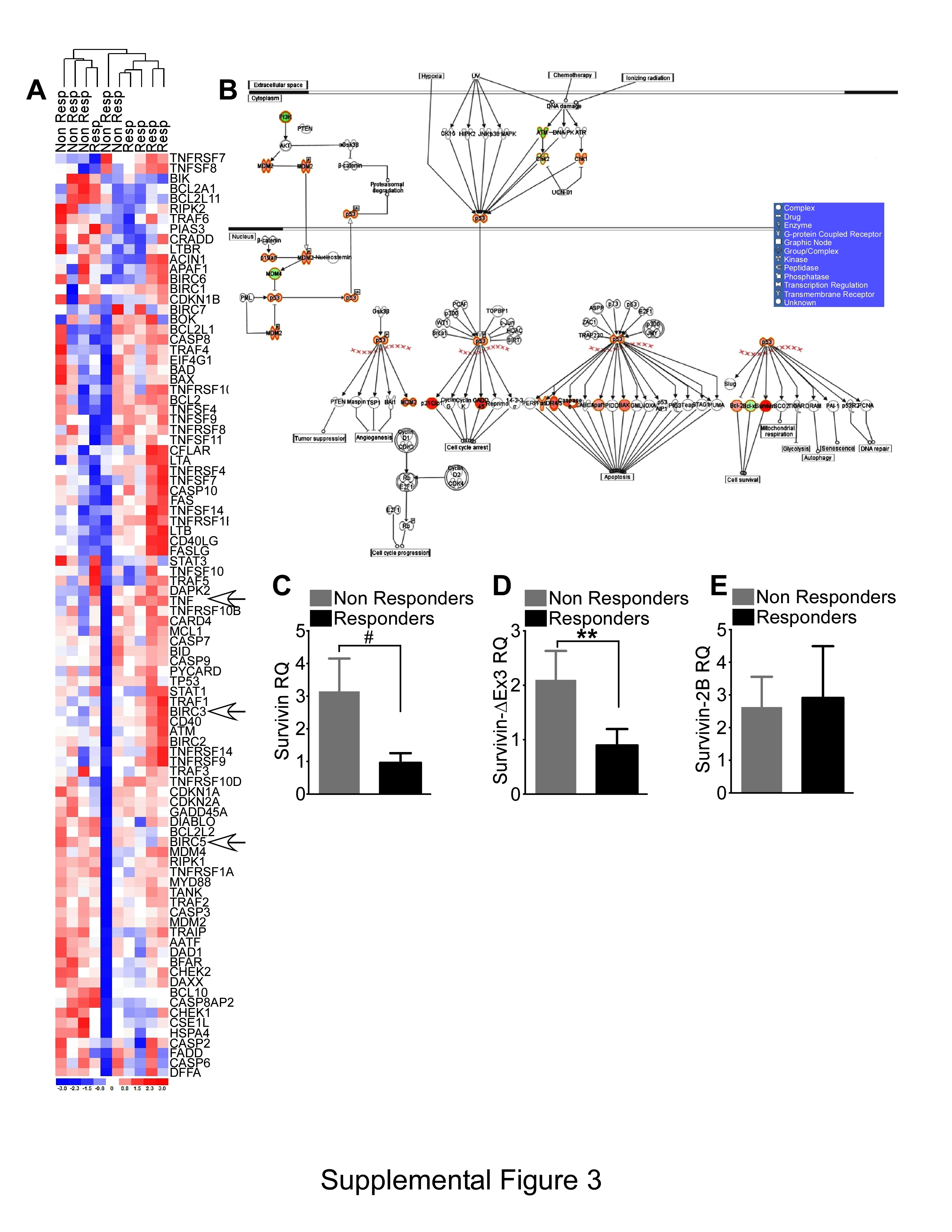


**Figure S3.** Differential expression of survivin family proteins in organotypic breast tumors. A, Heat map of differential expression of ninety-two apoptotic regulatory genes (Table S2) in Responder *versus* Non Responder *ex vivo* breast tumors (five cases per group) at baseline conditions. Arrows indicate the most differentially expressed genes between the two subgroups. B, Ingenuity pathway analysis of apoptotic gene profiling in Responder *versus* Non Responder breast tumors, as described in A. Up- or down-regulated genes in Non Responder tumors are indicated in red or blue, respectively. C-E, Expression of survivin (C) or splice variant survivin-ΔEx3 (D) or survivin-2B (E) in Non Responder (n=12) or Responder (n=17) breast tumors by qPCR. ^#^, *P*= 0.008; **, *P*=0.006 (unpaired *t* test).


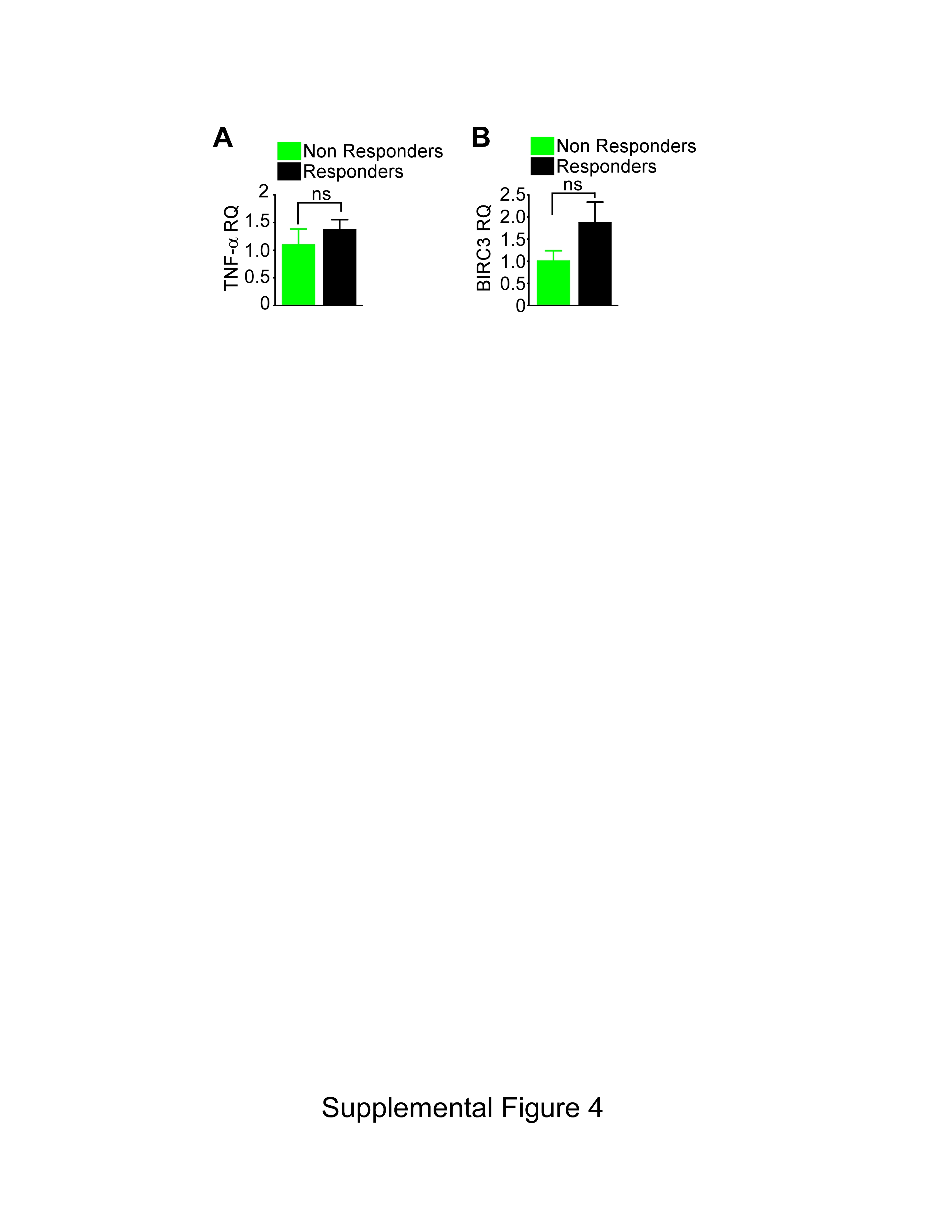


**Figure S4.** Expression of apoptosis regulators in organotypic breast tumors. The expression levels of TNF-α (A) or BIRC3 (B) genes was investigated in the complete series of organotypic breast tumors in response to doxorubicin treatment (n=29). Bars, mean±SEM.


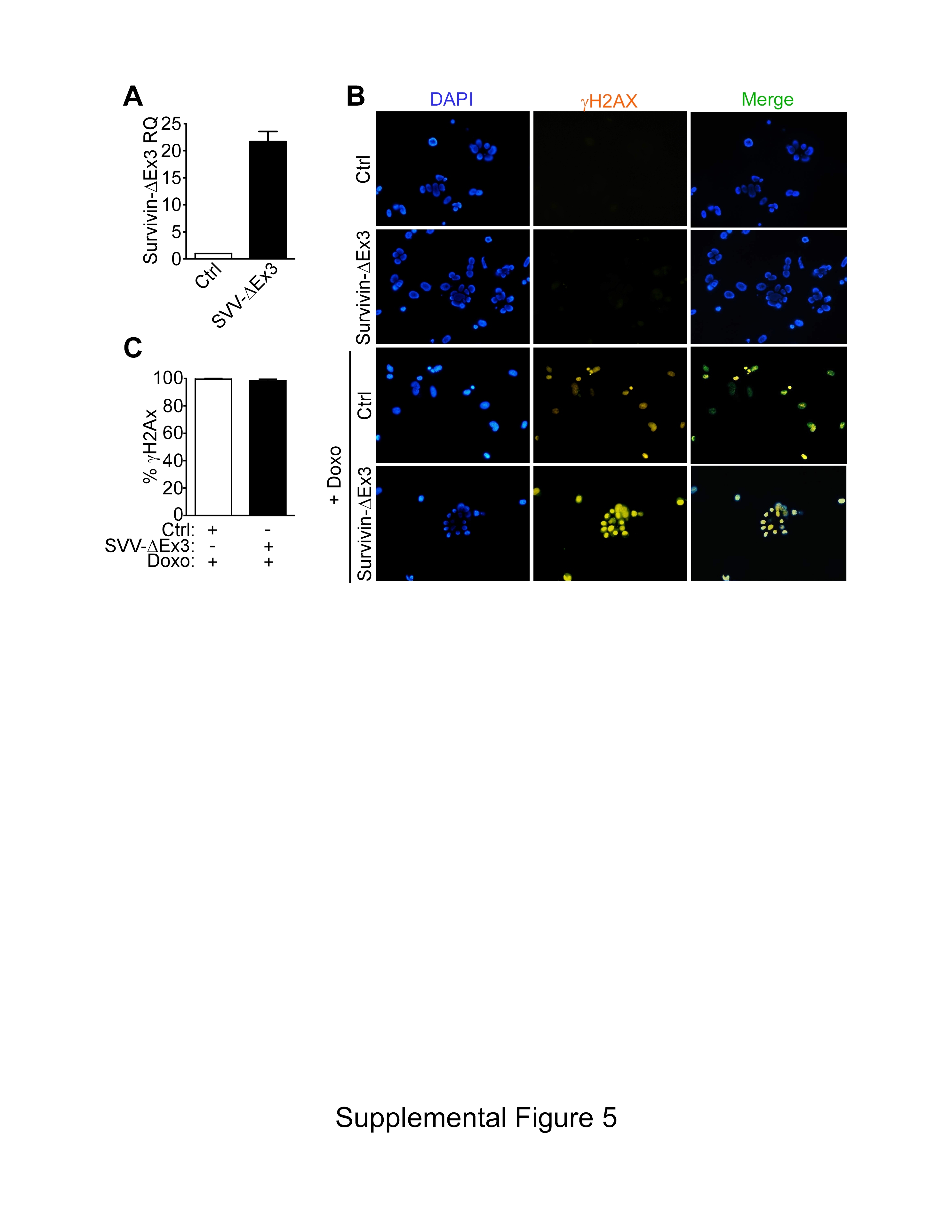


**Figure S5.** Effect of Survivin-ΔEx3 on the DNA-damage response. A, MCF-7 cells were transiently transfected with control plasmid (Ctrl) or Survivin-ΔEx3 (SVV-ΔEx3) and survivin-ΔEx3 mRNA expression was quantified after 48 h by qPCR. B, MCF-7 cells transfected as in A were analyzed for changes in phosphorylation of Histone H2AX (γH2Ax) by immunofluorescence in the presence or absence of 1 μM doxorubicin treatment (+Doxo) for 24 h. Original magnification, x200. C, quantification of γH2Ax-positive cells (%) from three independent experiments. Bars, mean±SEM.

1. ^a^ http://www.ncbi.nlm.nih.gov/gene [↑](#footnote-ref-2)
2. ^b^ Adapted from GeneCards V3 Human Genes Database; <http://www.genecards.org/> [↑](#footnote-ref-3)
3. Survivin isoform 1 was a commercially available assay from Applied Biosystems, LifeTechnologies. [↑](#footnote-ref-4)
